# ‘I am more stressed if my infection affects others’: development of a COVID-19-related stress scale in older people and examination of its validity and associations with mental health risks

**DOI:** 10.1192/bjo.2024.769

**Published:** 2024-10-25

**Authors:** Tianyin Liu, Lesley Cai Yin Sze, Eric Kwok Lun Yiu, Edwin Lok Yan Wong, Dara Kiu Yi Leung, Wai-wai Kwok, Jennifer Tang, Jiaqi Xu, Gloria Wong, Terry Lum

**Affiliations:** Department of Applied Social Sciences, The Hong Kong Polytechnic University, Hong Kong; Counseling and Wellness Center, The Hong Kong University of Science and Technology, Hong Kong; Department of Social Work and Social Administration, The University of Hong Kong, Hong Kong; School of Psychology, University of Birmingham, UK; Department of Educational Psychology, The Chinese University of Hong Kong, Hong Kong; Department of Psychology, The University of Hong Kong, Hong Kong

**Keywords:** Mixed-methods, bottom-up approach, measurement, COVID-19, mental health

## Abstract

**Background:**

COVID-19 was a collective traumatic event; however, different individuals may have perceived it differently.

**Aims:**

This study investigated what older people in a collective culture perceived as stressful during COVID-19 and examined how different stressors related to COVID-19 infection and mental health risks.

**Method:**

Thirty-six participants from diverse backgrounds engaged in a three-round Delphi study to generate items for a COVID-19-related stress scale for older adults (CSS-OA). Subsequently, 4674 people (aged ≥60 years) participated in a cross-sectional telephone survey; interviewers collected their responses to CSS-OA and information about COVID-19 infection, depressive symptoms, anxiety, loneliness and demographics. Exploratory factor analysis and confirmatory factor analysis were conducted on CSS-OA. A multiple indicator multiple cause (MIMIC) model was used to examine associations between CSS-OA and other measures.

**Results:**

The Delphi process generated eight items, all secondary or tertiary stressors related to infection. Exploratory factor analysis revealed a three-factor model, and confirmatory factor analysis confirmed an excellent fit (comparative fit index = 0.99, root mean square error of approximation = 0.06). Pre-existing mental health conditions, having family members/friends infected with COVID-19, loneliness, anxiety and depressive symptoms were associated with higher stress. Conversely, self-infection with COVID-19, older age, being female and living alone were negatively associated with some domains of CSS-OA (all *P* < 0.05).

**Conclusions:**

The Delphi process enhanced our understanding of what older people perceived as stressful, much of which resulted from certain healthcare strategies and reflected cultural influences. These and the MIMIC results highlight the need to balance public health policies with respect to infectious diseases and older people's mental health and quality of life.

COVID-19 outbreaks erupted in most nations in March 2020, negatively affecting populations worldwide.^1^ With the development of effective COVID-19 vaccines and increasing rates of vaccination,^2^ there has been a decreasing trend of COVID-19-related severe illness and death, and the World Health Organization announced that COVID-19 was no longer a global health emergency on 4 May 2023.^3^ Despite reductions in infection, severity, hospital admissions and mortality,^2^ COVID-19 is nevertheless a collective traumatic event worldwide that has adversely affected people's emotional well-being.^4^ Many COVID-19-related factors may contribute to this widespread adverse mental health impact, including active infection with the severe acute respiratory syndrome coronavirus 2 (SARS-CoV-2), the strain of coronavirus that caused COVID-19;^5^ the disease mitigation measures;^6^ and life adjustments that people had to make, e.g. stockpiling food despite price rises and changing working patterns.^7^ These factors caused multifaceted psychological stresses, some of which may linger and affect long-term mental health.

Stress can be conceptualised as a transaction that is both a result of the cognitive appraisal of a situation (primary appraisal) and the resources available to cope with the stressor (secondary appraisal).^8^ Multiple factors, some relating to the stressor and others inherent to the individual, may influence the manifestation of stress in different individuals at different times. SARS-CoV-2 naturally mutates over time, and omicron was the latest variant with more spike mutations and higher transmissibility; fortunately, it was less pathogenic than previous variants and caused fewer severe symptoms and hospital admissions.^9^ Therefore, the source of stress in the third year of the pandemic may differ from that in the beginning.

After the initial outbreak, there was an increasing need for mental healthcare providers to understand individuals’ responses related to COVID-19 and, subsequently, a need for a valid measurement instrument.^10^ Under these circumstances, a COVID-19-related stress scale (CSS) was developed.^10^ The CSS has 36 items, with five factors: (a) danger and contamination fears, (b) fears about socioeconomic consequences, (c) xenophobia, (d) compulsive checking and reassurance-seeking, and (e) traumatic stress symptoms related to COVID-19.^10^ The CSS has since been adapted and validated in different cultures and populations, including the Swedish general population^11^ and Iranian patients with obsessive–compulsive and anxiety disorders.^12^ However, the content of the CSS was developed using a top-down professional-led approach. Little is known about the viewpoints of older people and the ability of the CSS to capture perceived COVID-19-related stress in the third year of the pandemic. The Delphi technique is a recognised approach for gathering and consolidating opinions from diverse stakeholders regarding a particular subject, making it particularly apt for emerging research topics,^13^ and it was used here to reflect bottom-up viewpoints.

Particular demographic and inherent factors may also affect individuals’ perception of a stressful event. People with collectivist values may be more concerned with the needs and interests of their immediate family and social circles than those from individualistic cultures.^14^ It is worth noting that most published studies of COVID-19-related stress were conducted in relatively individualistic cultures, whereas perspectives from collectivist cultures are underrepresented in the existing literature. In addition, people with pre-existing mental health conditions may suffer from more significant psychological distress and perceived stress.^15^ Younger age, being female and living alone have also been found to be associated with higher stress levels.^16^ Although these demographic factors are not modifiable, investigating how they are related to COVID-19-related stress may increase our knowledge about who is at higher risk, thereby enabling the design of more person-centred mental health services.

Given the research gaps, this study had two objectives: (a) to implement a bottom-up approach to understand what older people in a predominately collectivist culture perceived as stressful and develop a CSS-OA; and (b) to investigate how the stressors are associated with COVID-19 infection, pre-existing mental health issues, current common mental health risks and demographic risk factors.

## Method

This study had two phases: in phase I, we developed the CSS-OA using the Delphi method; in phase II, we conducted a cross-sectional study to validate the scale and examine the associations between CSS-OA score and mental health risks in older people. The phase II study followed STROBE (Strengthening the Reporting of Observational Studies in Epidemiology) guidelines for cross-sectional studies (see Supplementary Table 1 available at https://doi.org/10.1192/bjo.2024.769).^17^

### Phase I: Delphi method to develop the COVID-19-related stress scale

#### Design

The Delphi method is a bottom-up approach that involves panel members who respond to researchers’ questions and controlled feedback for multiple rounds, and the process terminates once a predefined consensus rate is reached.^13^ Past research has often employed percentage agreement or central tendency measures to arrive at such consensus.^13^ There is no definite cut-off for consensus rating, and we set the consensus cut-off to 60% based on a recent empirical study suggesting that this is sufficient.^18^ Details of the qualitative and quantitative research methods used are presented in the procedure section.

#### Participants

Thirty-six participants with differing expertise in mental health in old age were recruited and formed the expert panel. All panel members were recruited from a holistic mental health programme for older people in Hong Kong (JC JoyAge).^19^ The JoyAge programme provides non-pharmacological mental health services for older people at risk of or with depressive symptoms. The panel comprised two clinical psychologists, three social workers, 20 trained volunteers in mental health services (older adults aged 50 years and above who had received mental health training) and 11 older mental health patients. The patients were older adults aged 60 years and over who had depressive symptoms and had received services from the JoyAge programme.

#### Procedure

We conducted a modified three-round Delphi survey during the worst COVID-19 community outbreak in Hong Kong from February to March 2022 (referred to as ‘the fifth wave’). In round one, we conducted a qualitative survey asking participants: ‘What do you think are the stressors perceived by older adults during the fifth wave of COVID-19?’ Each participant was allowed to suggest up to five answers. The researchers developed a list of stressors through qualitative analysis and synthesis of the collected responses. Duplicated items were eliminated, and a list of unique items was generated.

In round two, we conducted a quantitative survey with items generated from round one. We asked the participants: ‘To what extent do you feel that the following stressors during COVID-19 are relevant to older adults?’ on a seven-point Likert scale from 1 (not applicable) to 7 (extremely relevant). Items with scores of 5 (moderately relevant) and above were categorised as stressful (Table 1). The researchers summarised the ratings for each item (the average score and the percentage of participants who scored the item as stressful) and reduced the list based on the predefined consensus rate.

**Table 1:** COVID-19-related stress scale for older adults (CSS-OA) developed through the Delphi method

To what extent do you feel that the following stressors during COVID-19 are relevant to older adults? 1, not at all relevant; 2, mostly not relevant; 3, slightly not relevant; 4, neutral; 5, moderately relevant; 6, very relevant; 7, extremely relevant		
Unique items generated from Delphi round 1	Average rating, mean (s.d.)	Percentage of participants who rated the item as relevant to older people (score ≥5)
Final selected items (sequence based on conceptual similarity from Delphi round 3)^a^		
**1. Suspension of community service** (e.g. shut-down of community centres, suspension of food delivery)	4.87 (1.55)	75.00
**2. Price rises** (including daily goods and personal protective materials)	5.13 (1.39)	77.78
**3. Daily routine interference** (e.g. restrictions on going out, lack of exercise)	5 (1.98)	77.78
**4. Confusing pandemic-related policies** (e.g. stay-at-home or central quarantine centre)	5.04 (1.58)	80.56
**5. Self-infection will burden families**	4.59 (2.48)	63.89
**6. Families or friends infected with COVID-19**	4.57 (2.04)	66.67
**7. Need to visit hospital owing to other illnesses** (e.g. chronic disease, sudden fall)	4.50 (2.25)	69.44
**8. Concern for community members** (e.g. news reports about community members infected and dire consequences)	4.57 (1.85)	66.67
Other items not included in CSS-OA		
9. Unsure when COVID-19 will come to an end	4.55 (1.70)	58.33
10. Too much misinformation about COVID-19	4.52 (1.53)	58.33
11. Negative news about COVID-19-related consequences	4.38 (1.31)	58.33
12. Confusing information about the COVID-19 vaccine	3.96 (1.89)	55.56
13. Shortage of daily necessities and food supplies	4.26 (1.79)	52.78
14. Negative news report about the COVID-19 vaccine	4.43 (1.73)	50.00
15. Strict COVID-19 social distancing rules	4.29 (1.58)	47.22
16. Shortage of personal protective equipment against COVID-19	3.65 (1.64)	44.44
17. Role as a family carer	4.30 (1.99)	41.67
18. Fear of being infected with COVID-19	3.74 (2.28)	36.11
19. Feeling lonely	3.57 (1.90)	36.11
20. Side-effects of COVID-19 vaccines	3.78 (1.65)	36.11
21. The anxious atmosphere in the neighbourhood	3.52 (1.86)	36.11
22. Decreasing family income	3.65 (2.17)	33.33
23. Difficulty in booking a timeslot for vaccination	3.83 (1.64)	27.78
24. Feeling there is nothing one can do to combat COVID-19	3.35 (1.70)	25.00

a. The phase II telephone survey used items 1–8 (bold type).

In round three, participants were shown the results from round two for feedback. They were consulted on the face validity of the items, the wording and the response options. Throughout each round of Delphi, we primarily employed the survey platform Qualtrics to gather data, and we occasionally conducted interviews over the phone or face-to-face to suit the needs of participants with limited literacy.

#### Data analysis

Three researchers with psychology or social science training backgrounds conducted the analysis, summarised unique items, ranked participants’ ratings, consolidated feedback and finalised the Likert scale. At least two researchers worked on each step to reduce personal biases, and differences were resolved through group discussion and decision-making. One senior researcher oversaw all processes and work. Quantitative data analysis of the rating was conducted using SPSS (version 26).

### Phase II: rolling telephone survey

#### Design

Non-governmental organisations (NGOs) providing aged care or mental health services coordinated telephone screening to identify cases of potential mental health issues for early intervention among their members. All participating NGOs were partners in the JC JoyAge programme.^19^ The aged care and mental healthcare NGOs were both community-based service units where older adults received JoyAge services. Social workers or trained volunteers from NGOs conducted the cross-sectional telephone survey between April and July 2022.

#### Respondents

Respondents aged 60 years and above were recruited from 21 aged care and 13 mental healthcare NGOs in Hong Kong. Any community residents aged 60 and over are eligible to join aged care units, whereas only those with a mental illness history can join mental healthcare units. The interviewers contacted 4987 older adults who were existing users of the NGOs’ services, of whom 4674 responded to the screening (response rate 93.7%). All participants provided verbal consent before answering any question.

#### Measures

Respondents were interviewed following a protocol developed by two clinical psychologists and two researchers. The interview protocol comprised a warm greeting, verbal consent-taking and a questionnaire.

##### COVID-19 related stress

The COVID-19-related stress scale for older adults (CSS-OA) developed in phase I was used to assess respondents’ stress in different areas. Eight items were included in the CSS-OA based on preset criteria. Each item was rated on a six-point Likert scale based on how often the respondent felt stressed by the described events in the past 2 weeks, from 0 (not at all) to 5 (all the time). A rating of 3 or above indicates more than often.

##### Mental health risks screening

The Patient Health Questionnaire-2 (PHQ-2), Generalized Anxiety Disorder-2 (GAD-2) and Three-item Loneliness Scale (UCLA-3) were used for screening depressive symptoms, anxiety symptoms and loneliness, respectively. The PHQ-2 is a validated quick screening tool for major depressive disorders that uses the cardinal questions from the PHQ-9: anhedonia and depressed mood.^20^ The score for each question ranges from 0 (not at all) to 3 (nearly every day), and the validated Chinese version was used.^21^ GAD-2 is a screening tool for generalised anxiety disorder using the two core questions from the GAD-7 – feeling nervous/anxious and cannot control worrying – with scores ranging from 0 (not at all) to 3 (nearly every day) for each question.^22^ The validated Chinese version was used.^23^ UCLA-3 assesses the subjective feeling of loneliness by asking how often a person feels (a) lack of companionship, (b) left out and (c) isolated.^24^ A validated Chinese version was used with scores ranging from 0 (not at all) to 3 (nearly every day).^25^ For all individual items of PHQ-2, GAD-2 and UCLA-3, a cut-off score of 2 was applied to indicate more than half of the days being affected by the described problem; for total scores of each scale, a cut-off of 3 was applied for screening purposes.^21,23,25^

##### COVID-19 infection history

Two questions with a ‘yes/no’ response asked whether respondents (a) had been or were currently infected with COVID-19 themselves and (b) had family members and/or close friends who were infected.

##### Demographics

Age (years), sex (male versus female), living status (living alone versus living with other(s)), and NGO type (aged care versus mental healthcare, with the latter as a proxy for having a pre-existing mental health condition).

### Data analysis

First, descriptive statistics were used to summarise demographic variables and responses to all measures. Second, the data were randomly split into two files, a calibration file containing data from 2337 respondents and a validation file comprising data from 2337 respondents. Exploratory factor analysis (EFA) with Geomin rotation was performed to investigate the dimension of the eight-item CSS-OA using the calibration data-set. Geomin rotation was chosen because it is an oblique type of rotation that considers correlations between the factors, and it is advantageous in cases of variable complexity greater than one.^26^ Third, we performed confirmatory factor analysis (CFA) on the validation file driven by the EFA results. Fourth, we used multiple indicators multiple causes (MIMIC) modelling to measure the associations of CSS-OA domains with PHQ-2, GAD-2, UCLA-3, COVID-19 infection history, pre-existing mental health conditions and demographic variables. The MIMIC model is a specific application of the structural equation model that involves latent variables with multiple indicators that are simultaneously predicted by observed variables.^27^ The model fit of the EFA, CFA and MIMIC models was determined with the following commonly used indicators: χ^2^/d.f. ratio, comparative fit index (CFI), Tucker–Lewis Index (TLI) and absolute measure root mean square error of approximation (RMSEA). The usual criteria for an acceptable model fit are a χ^2^/d.f. ratio equal to or below 5, CFI and TLI values greater than 0.90, and RMSEA values below 0.08.^28^ Statistical analyses were conducted using SPSS (version 26.0), Mplus (version 8.3), and AMOS (version 24.0).

### Ethics statement

The authors assert that all procedures contributing to this work comply with the ethical standards of the relevant national and institutional committees on human experimentation and with the Helsinki Declaration of 1975, as revised in 2008. All procedures involving human subjects/patients were approved by the Human Research Ethics Committee of the University of Hong Kong (reference number: EA220106).

## Results

### Phase I: Delphi method results

Among the 36 participants in the Delphi study, five professionals, 18 trained volunteers and six older mental health patients completed all three rounds through the online platform; two trained volunteers and three patients participated through telephone interviews; and two patients participated through face-to-face interviews. The online group had better literacy than the telephone and face-to-face groups, but the items proposed by the three groups had large overlaps and no apparent differences in stress domains. Round one generated 66 raw items, with 24 unique items after removal of duplicates. In round two, eight items reached consensus based on pre-set criteria. In round three, participants checked the face validity of the eight items from round two; they suggested adding examples, using colloquial words, and adopting a six-point Likert scale based on the frequency of feeling stressed by the described factors in the past 2 weeks from 0 (not at all) to 5 (all the time) to avoid modesty answers (i.e. neutral point of the Likert scale – half of the time). Therefore, item response ≥3 on CSS-OA indicated feeling stressed for more than half of the time. The presented sequence of the final CSS-OA items was based on suggestions from clinical psychologists on conceptual similarity. Table 1 summarises the details of all 24 unique items and the consensus data.

### Phase II: telephone survey results

#### Descriptive statistics

Table 2 shows the descriptive statistics for all respondents (*N* = 4674) and separately for those without (*N* = 3878) and with pre-existing mental health conditions (*N* = 796). The total percentage of missing data was 3.05%; as this value was lower than 5%, we only used completed case data in the subsequent analyses.^29^ Respondents had a mean age of 75.63 years (s.d. = 8.87), 75.6% were female, 37.5% lived alone and 17% had pre-existing mental health conditions.

**Table 2:** Participants’ demographic and clinical characteristics (*N* = 4674)

	Total (*N* = 4674)		No pre-existing mental health condition (*N* = 3878)		With pre-existing mental health condition (*N* = 796)	
	Mean (s.d.)	*N* (%)	Mean (s.d.)	*N* (%)	Mean (s.d.)	*N* (%)
Basic demographics						
Age, years	75.63 (8.87)		76.83 (8.62)		69.79 (6.78)	
Sex, female		3531 (75.61)		2905 (74.9)		626 (78.6)
Living alone		1701 (37.51)		1395 (36.0)		306 (38.4)
Covid-19 infection history						
Family members/friends’ infection		1433 (30.88)		1107 (28.5)		326 (41.0)
Self-infection history		857 (18.34)		678 (17.5)		179 (22.5)
Response to scales		≥Cut-off		≥Cut-off		≥Cut-off
PHQ-2 total score, 0–6	1.02 (1.54)	654 (14.0)	0.82 (1.37)	401 (10.3)	1.96 (1.89)	253 (31.8)
PHQ-1 anhedonia, 0–3	0.54 (0.84)	613 (13.12)	0.44 (0.76)	373 (9.6)	0.76 (1.05)	240 (30.2)
PHQ-2 depressed mood, 0–3	0.48 (0.80)	548 (11.73)	0.38 (0.72)	342 (8.8)	0.72 (1)	206 (25.9)
GAD-2 total score, 0–6	0.92 (1.48)	550 (11.77)	0.74 (1.31)	339 (8.6)	1.79 (1.87)	211 (26.5)
GAD-1 feeling nervous/anxious, 0–3	0.51 (0.80)	550 (11.77)	0.42 (0.73)	345 (8.9)	0.73 (0.99)	205 (25.8)
GAD-2 cannot control worrying, 0–3	0.41 (0.75)	458 (9.8)	0.33 (0.67)	281 (7.2)	0.67 (0.98)	177 (22.2)
UCLA-3 total score, 0–9	1.57 (2.36)	1339 (28.65)	1.28 (2.11)	927 (23.9)	2.99 (2.93)	412 (51.8)
UCLA-1 lacking companionship, 0–3	0.56 (0.86)	720 (15.41)	0.46 (0.78)	455 (11.73)	1.06 (1.07)	265 (33.29)
UCLA-2 feeling left out, 0–3	0.46 (0.80)	577 (12.35)	0.37 (0.71)	353 (9.1)	0.93 (1.03)	224 (28.14)
UCLA-3 feeling isolated or lonely, 0–3	0.55 (0.88)	739 (15.81)	0.45 (0.79)	469 (12.09)	1.05 (1.1)	270 (33.92)
CSS-OA total score, 0–40	10.36 (9.71)		9.17 (9.14)		15.91 (10.29)	
CSS-OA individual items		Item response ≥3		Item response ≥3		Item response ≥3
Suspension of community services, 0–5	0.96 (1.28)	624 (13.35)	0.84 (1.19)	416 (10.7)	1.53 (1.51)	208 (26.1)
Price rises, 0–5	1.24 (1.49)	985 (21.07)	1.12 (1.43)	706 (18.2)	1.83 (1.62)	279 (35.1)
Daily routine interference, 0–5	1.20 (1.41)	886 (18.96)	1.06 (1.33)	612 (15.8)	1.86 (1.61)	274 (34.4)
Confusing pandemic-related policies, 0–5	1.35 (1.57)	1134 (24.26)	1.2 (1.49)	806 (20.8)	2.11 (1.72)	328 (41.2)
Self-infection will burden families, 0–5	1.44 (1.64)	1223 (26.17)	1.3 (1.57)	871 (22.5)	2.15 (1.8)	352 (44.2)
Family members/friends infected, 0–5	1.39 (1.60)	1162 (24.86)	1.23 (1.52)	804 (20.7)	2.19 (1.75)	358 (45.0)
Hospital visits due to other illnesses, 0–5	1.29 (1.56)	1074 (22.98)	1.13 (1.47)	744 (19.2)	2.09 (1.73)	330 (41.5)
Concern for community members, 0–5	1.46 (1.61)	1230 (26.32)	1.31 (1.55)	892 (23.0)	2.17 (1.71)	338 (42.5)

CSS-OA: COVID-19-related stress scale for older adults; GAD-2: Generalized Anxiety Disorder-2; PHQ-2: Patient Health Questionnaire-2; UCLA-3: UCLA three-item loneliness scale.

Reliability analyses suggested that all measures had good internal consistency (PHQ-2: Cronbach's α = 0.85; GAD-2: 0.89; UCLA-3: 0.92; CSS-OA: 0.92). Table 2 summarises participants’ scores on all measures. Based on validated cut-off scores, 654 (14.0%) respondents showed depressive symptoms, 550 (11.77%) showed anxiety symptoms, and 1339 (28.65%) felt lonely. In terms of perceived stress caused by various factors, ‘concern for community members’ (mean = 1.46, s.d. = 1.61) and ‘self-infection will burden families’ (mean = 1.44, s.d. = 1.64) had the highest ratings among the eight items.

#### Factor analyses of CSS-OA

Supplementary Table 2 shows the model-fitting indices of three EFA models using a randomly split half dataset (*N* = 2337) and the CFA based on the best model from EFA. Supplementary Table 3 summarises the factor loadings of the EFA models. The one-factor model did not have a satisfactory model fit (RMSEA = 0.14, TLI < 0.90); however, the two-factor and three-factor models both had satisfactory model fit. The Akaike information criterion (AIC) and Bayesian information criterion (BIC) of the three-factor model were lower than those of the two-factor model, indicating a better fit (AIC = 57224.60, BIC = 57437.60). The chi-squared test between the two-factor and three-factor models showed a significant difference (χ*^2^* = 125.64, *P* < 0.001), further supporting the three-factor model.

**Fig. 1:**
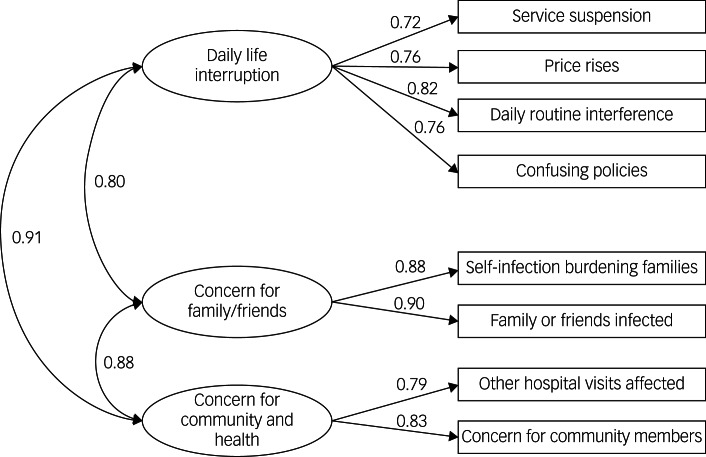
Confirmatory factor analysis standardised three-factor model of CSS-OA.

We then performed CFA with the validation sample, and the model fit indices were good (χ^2^/d.f. = 13.45, RMSEA = 0.06, CFI = 0.99, TLI = 0.98). Although the χ^2^/d.f. ratio was greater than 5, this was probably owing to the large sample size; all other model fitting indices suggested good fitting. Figure 1 summarises the standardised three-factor model parameter estimates. The three factors were labelled ‘Daily life interruption’ (DLI), ‘Concern for families/friends’ (CFF) and ‘Concern for community and health’ (CCH).

#### MIMIC model

**Fig. 2:**
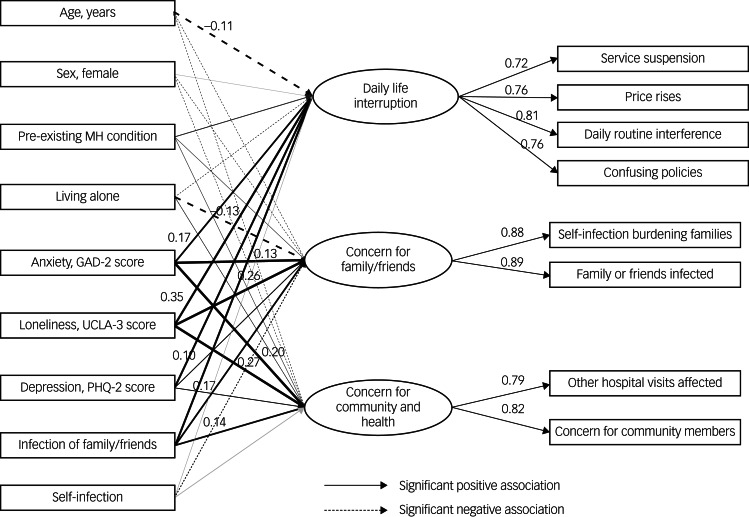
Multiple indicator multiple cause (MIMIC) models for three factors of COVID-19-related stress for older people, anxiety, loneliness, depression, COVID-19 infection history and demographics (*N* = 4674). Only standardised regression weights significant >0.10 at the *P* < 0.001 level are reported and highlighted; unstandardised regression weights and measurement errors were omitted for clarity. GAD-2: Generalized Anxiety Disorder-2; MH, mental health; PHQ-2: Patient Health Questionnaire-2; UCLA-3: UCLA three-item loneliness scale.

Table 3 shows the model-fitting indices of the MIMIC model and the associations between CSS-OA factors and other measures. The results of the MIMIC model suggested good model fitting (χ^2^/d.f. = 10.91, RMSEA = 0.05, CFI = 0.98, TLI = 0.96). Figure 2 summarises the standardised regression weights of significant associations greater than 0.1 between key variables under examination (see Supplementary Figure 1 for details).

**Table 3:** Regression weights and model-fitting indices for the multiple indicators multiple causes (MIMIC) model (*N* = 4674)

Pathways		β	*P*-value
Measurement model: COVID-19-related stress			
Daily life interruption (DLI)	Service suspension	0.72	<0.001
	Price rise	0.76	<0.001
	Daily routine interference	0.81	<0.001
	Confusing policies	0.76	<0.001
Concern for family members/friends (CFF)	Self-infection burdens families	0.88	<0.001
	Family members or friends infected	0.89	<0.001
Concern for community and health (CCH)	Other hospital visits affected	0.79	<0.001
	Concern for community members	0.82	<0.001
Factor intercorrelations			
DLI	CFF	0.73	<0.001
DLI	CCH	0.86	<0.001
CFF	CCH	0.85	<0.001
Structural model: COVID-19-related stress domains			
DLI	Age, years	−0.11	<0.001
	Sex, female	0.002	0.894
	Pre-existing mental health condition	0.06	<0.001
	Living alone	−0.04	0.007
	GAD-2	0.17	<0.001
	UCLA-3	0.35	<0.001
	PHQ-2	0.10	<0.001
	Family members/friends’ infection	0.09	<0.001
	Self-infection	−0.02	<0.001
CFF	Age, years	−0.07	<0.001
	Sex, female	−0.03	<0.001
	Pre-existing mental health condition	0.33	<0.001
	Living alone	−0.13	<0.001
	GAD-2	0.13	<0.001
	UCLA-3	0.26	<0.001
	PHQ-2	0.05	0.024
	Families/friends’ infection	0.17	<0.001
	Self-infection	−0.03	0.027
CCH	Age, years	−0.01	<0.001
	Sex, female	−0.05	<0.001
	Pre-existing mental health condition	0.09	<0.001
	Living alone	−0.05	0.003
	GAD-2	0.20	<0.001
	UCLA-3	0.27	<0.001
	PHQ-2	0.09	<0.001
	Family members/friends’ infection	0.14	<0.001
	Self-infection	−0.03	0.043
Model fit^a^			
χ^2^/d.f.		676.225/62 = 10.907	676.225/62 = 10.907
*P*-value		<0.001	<0.001
SRMR		0.021	0.021
RMSEA		0.046	0.046
CFI		0.982	0.982
TLI		0.955	0.955

CFI, comparative fit index; GAD-2, Generalized Anxiety Disorder-2; PHQ-2, Patient Health Questionnaire-2; RMSEA, root mean square error of approximation; SRMR, standardised root mean square residual; TLI, Tucker–Lewis index; UCLA-3, UCLA 3-item loneliness scale.

a. The criteria for a good model fit were: SRMR < 0.08, RMSEA < 0.08, CFI > 0.90, TLI > 0.90.

As shown in Table 3, depressive symptoms, anxiety and loneliness were all significantly associated with heightened stress for all three factors. Loneliness had the strongest association with CSS-OA for all three factors (β = 0.35, with DLI, 0.26 CFF, 0.27 CCH; all *P* < 0.001). Anxiety symptoms were positively associated with all three factors of CSS-OA (β = 0.17, with DLI, 0.13 CFF, 0.20 CCH; all *P* < 0.001). Depressive symptoms were significantly but weakly associated with CSS-OA (β = 0.10, with DLI, 0.05 CFF, 0.09 CCH; all *P* < 0.05). Regarding COVID-19 infection history, having infected family members/friends was positively associated with higher stress in all domains, most strongly with the CFF (β = 0.17, *P* < 0.001). Conversely, despite weak associations, self-infection was negatively related to higher stress in all domains (β = −0.02, with DLI, −0.03 CFF, −0.03 CCH; all *P* < 0.05). Having pre-existing mental health conditions was positively associated with higher stress, most strongly with CFF (β = 0.33, *P* < 0.001).

Finally, regarding basic demographics, age (DLI, β = −0.11; CFF, β = −0.07; CCH, β = −0.01; all *P* < 0.001) and living alone (DLI, β = −0.04, *P* < 0.05; CFF, β = −0.13, *P* < 0.001; CCH, β = −0.05, *P* < 0.05) were negatively associated with heightened stress in all three domains. Being female was negatively but weakly associated with stress in two domains (CFF, β = −0.03; CCH, β = −0.05; both *P* < 0.001).

## Discussion

This is the first study to use the Delphi technique to understand what older people in a Chinese community perceived as stressful during COVID-19. It thus augments the literature on perceived stress in a global health crisis with the previously largely neglected voices of older people from a collective culture. We also investigated how different stress domains were simultaneously associated with pre-existing mental health issues, concurrent common mental health problems, COVID-19 infection experiences and demographic risk factors. The results may help to identify groups at risk of mental health problems and inform mental health services.

First, we found unique stressors that were perceived as significant by older people in Hong Kong but considered to be less important or not reported in previous literature (e.g. the belief that self-infection will burden families, and concern for community members). Comparing CSS-OA with the original CSS, there were some overlaps; for example, ‘I am worried that I can't keep my family safe from the virus’ and ‘I am worried that grocery stores will close down’ from the CSS were similar to the CFF and DLI domains in CSS-OA.^10^ This may reflect a universal psychological response to a widespread public health crisis. Conversely, some stressors commonly reported in previous studies, such as fear of infection, received low consensus. These differences might be attributed to the variations in study contexts, particularly culture and timing. Consistent with the findings of a previous study that family members’ well-being was negatively associated with individuals’ psychological distress among Chinese participants,^30^ we also found that older Chinese people were stressed about their families becoming infected or about the burden of their own infection on families and friends. It is likely that when older adults were affected, their families and close friends would serve as caregivers, and that these individuals would have taken on increased responsibilities during the pandemic and would also have been under heightened stress. Moreover, such concern was extended to community members when the omicron variant caused more infections than previous community outbreaks, manifesting the values of a collectivist culture. Timing also mattered. Before the ‘fifth wave’, Hong Kong managed to contain the disease reasonably well; however, after initial success, stringent public health and social distancing measures extending over 2 years became a double-edged sword, and growing pandemic fatigue began to spread.^31^ Therefore, fear of infection was less of a concern, and interruption to daily life became the primary stressor.

Second, we found a three-factor structure of CSS-OA, endorsing the multifactorial nature of COVID-19-related stress. The multidimensional scales suggested a broader and more nuanced conceptualisation of stress in a global public health crisis. Our findings converged partially on the socioeconomic consequences (DLI: price rise) and danger (family-related items) domains of the original CSS. Stressors in these domains were not directly associated with SARS-CoV-2 self-infection, which emerged in different studies among different populations at different times of the pandemic. Therefore, we hypothesise that the stressors related to socioeconomic consequences in daily life and danger to family and close friends may extend to other populations and may last for longer than other stressors, even after the pandemic.

Third, we found that infection of family members and friends was associated with higher stress in all three domains of CSS-OA, but self-infection was weakly and negatively associated with stress. The heightened stress associated with family members’ and friends’ infection was logical and provided further evidence of the influence of collectivist culture on individuals’ value systems. Self-infection, on the other hand, was associated with lower stress, despite a weak association. This finding may be understood in terms of the uncertainty theory of anxiety, which postulates that reducing uncertainty may reduce anxiety, at least temporarily.^32^ When applied to this study, if the survey respondents had been infected with the virus, they would have either recovered and gained immunity or were physically capable of answering the phone call despite being infected. Their uncertainty regarding when they would be infected and what symptoms they would have would be reduced.

Fourth, having pre-existing mental health conditions and concurrently scoring higher on common mental health screening tools were associated with higher stress. When considered simultaneously, standardised results suggested that loneliness had the strongest association with the DLI and CCH domains, whereas having pre-existing mental health conditions was most strongly associated with CFF. Although Hong Kong never had a complete lockdown, strict social distancing measures were imposed, and older people were more likely to self-isolate and avoid crowds.^33^ Going to community aged centres was one of the primary channels by which older people could receive services and socialise with peers in Hong Kong. However, this channel was restricted during the pandemic except for limited online services, leaving older people more socially isolated because of low digital literacy;^34^ this explains the association between the DLI domain and loneliness. Attending hospital for regular follow-ups was another motivation for older people to leave their home; however, it was reported in 2020 that after the initial outbreak, missed medical appointments over 3 months increased from 16.5% to 22.0% among older adults in Hong Kong,^35^ possibly explaining the association between the CCH domain stressor and loneliness. The finding that people with pre-existing mental health conditions were more vulnerable to stressors was consistent with previous findings.^36^ As stress is usually temporary, mentally healthy individuals may bounce back after the removal of the stress, but this may not be the case for people with mental health conditions. The strongest association with the CFF domain may have been owing to higher reliance on family/friends as informal caregivers among this population; hence, individuals were more sensitive about the additional burden that they might cause. To build resilience, especially for older adults with mental illness history, population-based approaches to mental healthcare should be adopted, incorporating different degrees of preventive strategies and promoting cross-sector collaboration.^37^ In the community setting, non-clinical interventions and activities for improving mental health outcomes could be offered to older adults and their caregivers. Activities that may enhance adaptive coping strategies, such as seeking social support and problem-solving orientation, are especially relevant during COVID-19.

Finally, older age, living alone and being female were all negatively associated with higher COVID-19-related stress, in contrast to the findings of some previous studies.^16^ The negative association between older age and pandemic-related stress is consistent with an increasing number of studies demonstrating the resilience of older people during the pandemic.^38^ Living alone was negatively associated with COVID-19-related stress, especially in the CFF domain; this was reasonable, because even if older people became infected, they would not immediately pass the infection to family members. Being female was negatively associated with stress in the CFF and CCH domains. We speculate that these results might have been driven by the disproportionate sex ratio in our sample; the effects of being female on perceived stress were attenuated when the effects of multiple causes were examined simultaneously.

### Strengths and limitations

This study had several strengths. First, we used a systematic bottom-up approach to understand what older people perceived as stressful and cross-validated the results with mental health professionals. This approach enabled us to be more grounded and go beyond the conventional professional-led framework in scale development. Second, we recruited a large sample of community-dwelling older people in the telephone survey. The high response rate of 93.7% suggested that the NGOs had good engagement with and enjoyed the trust of community members, and that older people were invested in responding to the questions. It may also indicate that older people felt lonely during COVID-19 and needed more community care, even just a phone call. Third, the MIMIC model enabled us to simultaneously evaluate the effects of the covariates on the factor indicators, providing better insight into the relationships between measured items, latent variables and covariates.

Several limitations should be noted when interpreting the findings. First, because of the cross-sectional design and convenient sampling, no causal relationships can be drawn, and the study results cannot be generalised to the general older adult population. Second, dependence on a telephone survey precluded participants with severe hearing loss or without access to a telephone. Third, we used membership of mental health service centres as a proxy for having a pre-existing mental health condition but did not ask respondents for self-report or any diagnosis. Older adults involved with aged care centres may also have mental illness but not seek help because of low awareness, lack of knowledge or stigma about mental illness. Finally, this study only included a few demographic risk factors; we did not collect information about other crucial factors such as physical health. Future investigations could consider longitudinal designs with repeated measures of common mental health risks and could include more documented risk factors and questions about mental illness history. For example, more targeted interventions on loneliness and for those with pre-existing mental health conditions could be designed, such as online support groups and teletherapy, and we could use CSS-OA to evaluate the effectiveness of these interventions on perceived stress. We may also conduct longitudinal research to track changes in stress levels among older adults over time using CSS-OA, following up with the respondents in this study from the peak of the pandemic to the post-pandemic period, which could help to identify factors that contribute to resilience or prolonged stress in this population.

### Implications

This study updates our understanding of what older people perceive as stressful in a prolonged pandemic. Using a bottom-up approach, we learned that older people in a collectivist society were concerned about how their infection might burden families and friends and gained insight into how COVID-19 and related healthcare strategies affected older people's daily lives. The differential associations among pre-existing mental health conditions, infection of family members and/or friends, concurrent loneliness risks, anxiety, depressive symptoms and stress may have service implications. In a public health crisis such as COVID-19, both older adults and their informal caregivers are likely to require help to deal with amplified stress; therefore, public assistance, including physical and mental health services, could target families or even the community as service units. More attention could be given to those with pre-existing mental health conditions, for example, empowering older adults with a history of common mental health conditions and their informal carers with adaptive coping strategies. To increase the population's resilience to stress, preventive mental healthcare could be adopted; this could include universal prevention for all, selective prevention for those with risk factors and indicated prevention for those with symptoms detectable through quick screening.

Based on the current survey results, social service sectors could consider providing interventions to target loneliness and building networks to reduce perceived stress among older people. One plausible approach could involve implementing interventions designed to enhance social connectivity and relationships; for example, cognitive–behavioural therapy, which has been validated as an effective measure of ameliorating loneliness.^39^ Furthermore, digital interventions have been explored with respect to their capacity to mitigate loneliness, and existing interventions facilitated through technology, such as online interactive modules, have been evaluated as both feasible and acceptable.^40^ These technological interventions present a readily accessible and convenient avenue for providing support and mitigating loneliness, particularly in times of social isolation.

It is worth noting that the stressors achieving high consensus were all secondary or tertiary to infection, and many may emanate from healthcare strategies for controlling infectious diseases. Future disease mitigation measures and public health policies need to balance the need for disease control with people's mental health and quality of life. Clear and transparent communication is needed, and public health measures need to be tailored to the severity of the disease in specific regions, allowing for more localised responses. Targeted support, including ensuring access to healthcare, social services and financial assistance, should be provided to vulnerable populations who may be affected disproportionately by disease and mitigation measures.

Finally, the CSS-OA scale could be used for more than monitoring the stress levels of older adults during and after COVID-19. Owing to climate change, urbanisation, globalisation and many other factors, the likelihood of future public health crises is high, and the CSS-OA could be used or adapted to gain insights into older people's stress levels and mental health needs. Some of the items non-specific to the pandemic could be used to assess chronic or transmissible disease-related distress (e.g. being a burden to the family), but more research is needed to validate this use.

To sum up, the current study provides a bottom-up perspective on what older adults perceive as stressful and offers insight into associations with mental health risks during COVID-19, which could aid in designing future public health strategies. These strategies could specifically address the unique needs of older adults, enhancing resilience and promoting well-being during times of crisis.

## Supporting information

Liu et al. supplementary material Liu et al. supplementary material

## Data Availability

The data, analytical methods and materials supporting this study's findings are available from the corresponding author upon reasonable request.
