# Supplementary material for: ‘I am more stressed if my infection affects others’: development of a COVID-19-related stress scale in older people and examination of its validity and associations with mental health risks
Source: BJPsych Open. 2024 Oct 25;10(6):e187. doi: 10.1192/bjo.2024.769 (PMC11698184; doi:10.1192/bjo.2024.769)
Supplement: Liu et al. supplementary material [file S2056472424007695sup001.docx]

Supplementary Table 1. STROBE Statement — Checklist of items of Phase II cross-sectional telephone survey.

|  | Item No | Recommendation | Respected? | Comments and quotes |
| --- | --- | --- | --- | --- |
| **Title and abstract** | 1 | (*a*) Indicate the study’s design with a commonly used term in the title or the abstract | Yes | The study design is indicated in the Methods section of the abstract. |
|  |  | (*b*) Provide in the abstract an informative and balanced summary of what was done and what was found | Yes | This information is stated in the study abstract: aims, methods, and results described structurally |
| Introduction | | |  |  |
| Background/rationale | 2 | Explain the scientific background and rationale for the investigation being reported | Yes | The existing literature and rationales are stated in the introduction section |
| Objectives | 3 | State specific objectives, including any prespecified hypotheses | Yes | The objectives are specified at the end of the introduction section. “Given the research gaps, this study had two objectives: (1) implement a bottom-up approach to understand what older people in a predominately collectivist culture perceived as stressful and develop a COVID-19-related stress scale for older adults (CSS-OA); and (2) investigate how the stressors are associated with COVID-19 infection, pre-existing mental health issues, current common mental health risks and demographic risk factors.” |
| Methods | | |  |  |
| Study design | 4 | Present key elements of study design early in the paper | Yes | The study design is described in the first subsection of Methods, and key elements used in Phase II are described as well. |
| Setting | 5 | Describe the setting, locations, and relevant dates, including periods of recruitment, exposure, follow-up, and data collection | Yes | The setting, locations, and dates of telephone survey periods are fully described in the methods section under Phase II. |
| Participants | 6 | (*a*) Give the eligibility criteria, and the sources and methods of selection of participants | Yes | The study population is described in the methods section Phase II respondents. |
| Variables | 7 | Clearly define all outcomes, exposures, predictors, potential confounders, and effect modifiers. Give diagnostic criteria, if applicable | Yes | Standardized measurements address the outcomes, and they are clearly stated in the methods/phase ii/measures section. |
| Data sources/ measurement | 8* | For each variable of interest, give sources of data and details of methods of assessment (measurement). Describe comparability of assessment methods if there is more than one group | Yes | Data collection and measurement are the same for all respondents of the telephone survey and are described in the methods section. |
| Bias | 9 | Describe any efforts to address potential sources of bias | Yes | We notably tried to reduce bias in both Phase I and Phase II of the study. In Phase I, we recruited different stakeholders to balance the bottom-up views, and in the analysis, “at least two researchers worked on each qualitative step to reduce personal biases, and differences were resolved through group discussion and decision-making.” In Phase II |
| Study size | 10 | Explain how the study size was arrived at | Yes |  |
| Quantitative variables | 11 | Explain how quantitative variables were handled in the analyses. If applicable, describe which groupings were chosen and why | Yes |  |
| Statistical methods | 12 | (*a*) Describe all statistical methods, including those used to control for confounding | Yes |  |
|  |  | (*b*) Describe any methods used to examine subgroups and interactions | Yes |  |
|  |  | (*c*) Explain how missing data were addressed | Yes |  |
|  |  | (*d*) If applicable, describe analytical methods taking account of sampling strategy | Yes |  |
|  |  | (*e*) Describe any sensitivity analyses | N/A |  |
| Results | | |  |  |
| Participants | 13* | (a) Report numbers of individuals at each stage of study—eg numbers potentially eligible, examined for eligibility, confirmed eligible, included in the study, completing follow-up, and analysed | Yes |  |
|  |  | (b) Give reasons for non-participation at each stage | N/A |  |
|  |  | (c) Consider use of a flow diagram | N/A | Use of a flow diagram was not deemed necessary for the study’s nature |
| Descriptive data | 14* | (a) Give characteristics of study participants (eg demographic, clinical, social) and information on exposures and potential confounders | Yes | The information of the participants are summarized in Table 2 |
|  |  | (b) Indicate number of participants with missing data for each variable of interest | Mostly |  |
| Outcome data | 15* | Report numbers of outcome events or summary measures | Yes |  |
| Main results | 16 | (*a*) Give unadjusted estimates and, if applicable, confounder-adjusted estimates and their precision (eg, 95% confidence interval). Make clear which confounders were adjusted for and why they were included | Mostly | Non-adjusted estimates were not displayed in the interest of table clarity. |
|  |  | (*b*) Report category boundaries when continuous variables were categorized | Yes | Category boundaries are displayed in variable headings in the tables, where applicable |
|  |  | (*c*) If relevant, consider translating estimates of relative risk into absolute risk for a meaningful time period | N/A | N/A |
| Other analyses | 17 | Report other analyses done—eg analyses of subgroups and interactions, and sensitivity analyses | N/A | N/A |
| Discussion | | |  |  |
| Key results | 18 | Summarise key results with reference to study objectives | Yes | We summarised the key results from Phase I and Phase II to address the two objectives in the introduction. |
| Limitations | 19 | Discuss limitations of the study, taking into account sources of potential bias or imprecision. Discuss both direction and magnitude of any potential bias | Yes | Description of limitations is done under “Strengths and limitations” heading in the discussion |
| Interpretation | 20 | Give a cautious overall interpretation of results considering objectives, limitations, multiplicity of analyses, results from similar studies, and other relevant evidence | Yes | References were added where appropriate and discussed. Limitations were taken into account in the discussion. |
| Generalisability | 21 | Discuss the generalisability (external validity) of the study results | Yes | We addressed in the discussion limitation section that “the study results cannot be generalised to the general older adult population”. |
| Other information | | |  |  |
| Funding | 22 | Give the source of funding and the role of the funders for the present study and, if applicable, for the original study on which the present article is based | Yes | Funding information was displayed upon submission. “This work is supported by the Hong Kong Jockey Club Charites Trust for The University of Hong Kong for the Project JC JoyAge: Jockey Club Holistic Support Project for Elderly Mental Wellness (HKU Project Codes AR160026, AR190017). ” |

Supplementary Table 2. Model-fitting indices of Exploratory Factor Analysis (EFA) and (Confirmatory Factor Analysis) CFA models

| Model fit Indices ^a^ | **EFA models** | | | **CFA** | |
| --- | --- | --- | --- | --- | --- |
|  | **One-factor** | **Two-factor** | **Three-factor** | | **Three-factor** |
| χ^2^ | 948.480 | 179.655 | 54.017 | | 174.847 |
| df | 20 | 13 | 7 | | 17 |
| P | <0.001 | <0.001 | <0.001 | | <0.001 |
| SRMR | 0.053 | 0.021 | 0.012 | | 0.015 |
| RMSEA | 0.141 | 0.074 | 0.054 | | 0.063 |
| 95% CI | 0.133 0.149 | 0.065 0.084 | 0.041 0.067 | | 0.055 0.072 |
| CFI | 0.918 | 0.985 | 0.996 | | 0.986 |
| TLI | 0.885 | 0.968 | 0.983 | | 0.977 |
| AIC | 58093.066 | 57338.241 | 57224.603 | | 57459.273 |
| BIC | 58231.225 | 57516.697 | 57437.598 | | 57614.702 |

AIC: Akaike information criterion; BIC: Bayesian information criterion; CFI: comparative fit index; *df:* degree of freedom; RMSEA: root mean square error of approximation; SRMR: standardized root mean square residual; TLI: Tucker-Lewis index; χ^2^: Chi-square.

^a^ The criteria for a good model fit are: SRMR < 0.08, RMSEA < 0.08, CFI > 0.90, TLI > 0.90.

Supplementary Table 3. Geomin rotated factor loadings of EFA models

| Items | One-Factor | Two-Factor | | Three-Factors | | |
| --- | --- | --- | --- | --- | --- | --- |
|  | 1 | 1 | 2 | 1 | 2 | 3 |
| CSS-old1 | 0.649* | 0.713* | -0.008 | -0.007 | 0.721* | -0.007 |
| CSS-old2 | 0.716* | 0.738* | 0.035 | -0.007 | 0.731* | 0.047 |
| CSS-old3 | 0.752* | 0.831* | -0.009 | -0.007 | 0.848* | -0.015 |
| CSS-old4 | 0.758* | 0.571* | 0.235* | 0.006 | 0.546* | 0.249* |
| CSS-old5 | 0.802* | 0.068 | 0.809* | -0.006 | 0.073 | 0.807* |
| CSS-old6 | 0.815* | -0.011* | 0.919* | -0.005 | -0.017 | 0.929* |
| CSS-old7 | 0.765* | 0.392* | 0.417* | 0.423* | 0.372* | 0.008 |
| CSS-old8 | 0.795* | 0.437* | 0.400* | 6.285* | 0.000 | 0.000 |
| Geomin factor correlations |  | r12=0.74 | - | r12=0.12 | - | - |
|  |  |  |  | r13=0.12 | r23=0.73 | - |

* p < 0.05. Salient (parameter estimates > 0.4 and significant) loadings are bold.


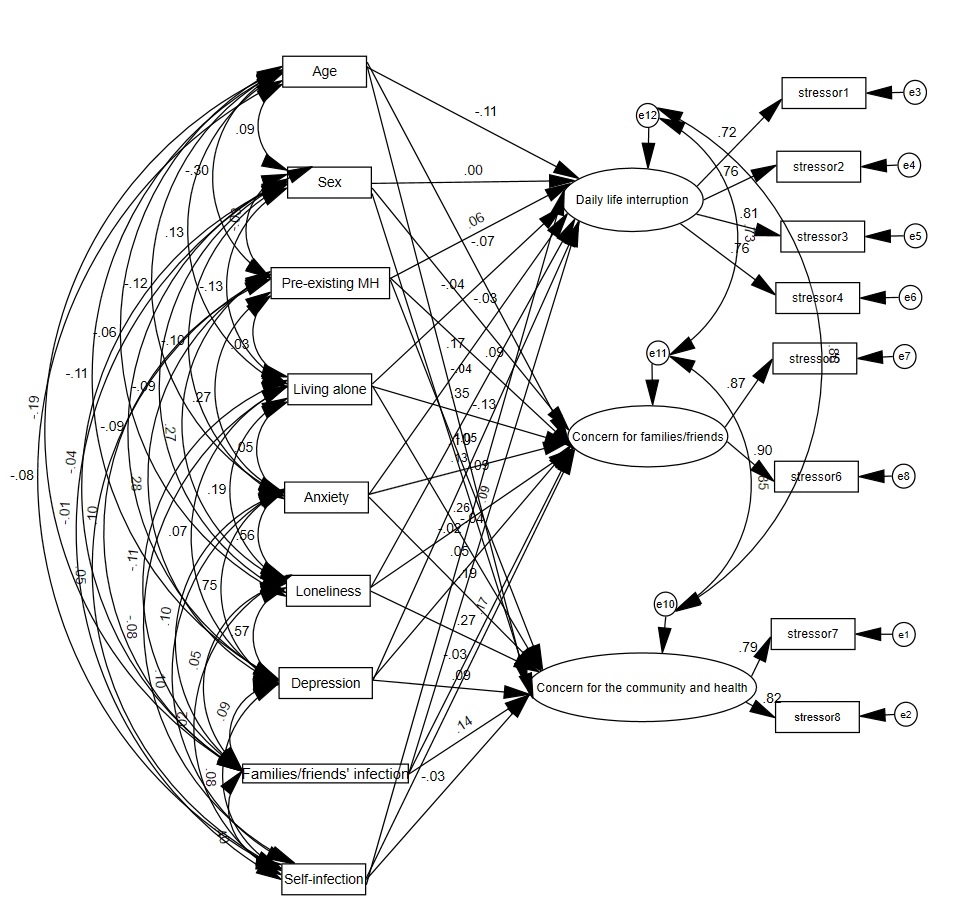


Supplementary Figure 1. Multiple indicator multiple cause (MIMIC) models for three factors of Covid-19-related stress for older people, anxiety, loneliness, depression, COVID-19 infection history and demographics (N = 4674).
